# Nationwide Survey of Oral Function Management in Patients With Neuromuscular Intractable Diseases in Japan

**DOI:** 10.7759/cureus.81770

**Published:** 2025-04-05

**Authors:** Mitsuyo Shinohara, Satomi Shiota, Yoshiko Yamamura, Shinpei Abe, Rie Shinohara, Kazuhisa Takahashi, Shunsuke Namaki, Nobutaka Hattori

**Affiliations:** 1 Department of Oral and Maxillofacial Surgery, Faculty of Medicine, Juntendo University, Tokyo, JPN; 2 Department of Respiratory Medicine, Faculty of Medicine, Juntendo University, Tokyo, JPN; 3 Department of Pediatrics, Faculty of Medicine, Juntendo University, Tokyo, JPN; 4 Department of Orthodontics, School of Dentistry, Nihon University, Tokyo, JPN; 5 Department of Respiratory Medicine, Graduate School of Medicine, Juntendo University, Tokyo, JPN; 6 Department of Oral and Maxillofacial Surgery, Nihon University School of Dentistry, Tokyo, JPN; 7 Department of Neurology, Faculty of Medicine, Juntendo University, Tokyo, JPN

**Keywords:** dental problems, japanese, muscular dystrophy, myasthenia gravis, myopathy, neuromuscular intractable disease, oral function management, periodontitis, questionnaire survey, spinal muscular atrophy (sma)

## Abstract

Aims: We investigated the current situation and problems associated with dental treatment for patients with neuromuscular intractable diseases at medical institutions in Japan.

Materials and methods: Questionnaires were sent to 658 dental medicine institutions nationwide. Between October 25 and December 31, 2021, we targeted dental hospitals, dental and oral surgery departments from university hospitals, and dental treatment facilities registered with the Japanese Society of Disability Dentistry that had pediatric dentistry, disability dentistry, or eating function therapy departments. A questionnaire survey was conducted on the following topics: whether or not the dental clinic had experience in treating patients with neuromuscular disorders, breakdown of diseases and annual number of patients, oral symptoms, treatment content, frequency of hospital visits, problems faced by patients and their families in terms of dental treatment, and points that dental healthcare professionals pay particular attention to when treating patients.

Results: Responses were received from 215 facilities. Muscular dystrophy was the most common disease affecting patients who were treated in the course of a year, accounting for approximately 40% of the total. The most common oral symptom was periodontitis, and the treatment for it consisted of oral care (including removal of tartar and plaque), performed in approximately half of the patients, followed by general dental treatment and swallowing training. Oral care was carried out regularly. The main problems faced by the patients were difficulties with self-care on the part of the patients and their families, and difficulties with attending hospital appointments due to a lack of social support. Lack of acceptance at local dental clinics was also mentioned. Treatment considerations that required particular attention from the medical staff were the most frequent, with the most common being adequate positioning to prevent aspiration and ensure appropriate suction. In addition, a third party was requested to accompany the patient when attending hospital appointments, and vital signs were checked during treatment.

Conclusion: Many patients with neuromuscular syndromes have oral diseases such as periodontitis and dental caries, as well as dysphagia, and require the intervention of dentists. However, dental professionals may not always be prepared to accept these patients. It is urgent for dentists to deepen their knowledge about the appropriate treatment for these patients and build a system capable of accepting them, while maintaining close cooperation with the medical profession.

## Introduction

Due to advances in medical technology, the average life expectancy of patients with neuromuscular diseases such as muscular dystrophy (hereafter, neuromuscular diseases) has increased dramatically in recent years [1,2]. Maintaining good mastication and swallowing functions, as well as oral hygiene, is extremely important for their normal development and to prevent infections. There is very limited research on the management of oral function in patients with neuromuscular disorders, both in Japan and overseas, and this has a significant impact on the quality of medical services. In particular, there is a lack of systematic surveys and empirical research on this issue in Japan, and an urgent need to develop a database that can be used in policy planning and clinical practice. However, although many of these patients have several dental problems such as reduced mastication and swallowing function, and difficulties with self-care due to their primary disease [3], there are a few reports on their clinical conditions both in Japan and overseas [4,5], and a few surveys conducted via questionnaire have been reported [6], with most of them focused on responses from patients and almost none on those from medical professionals.

Oral care as a means of supporting patient independence in daily life is important, and appropriate oral function management positively impacts participation in social activities and mental health. In this study, we conducted a questionnaire survey on the actual situation of patients with neuromuscular disorders and the problems they face regarding dental treatment at medical institutions across Japan. This survey aimed to clarify the current situation regarding oral function management in patients with neuromuscular disorders, propose specific measures that dentists should adopt based on this information, and promote the formulation of practical guidelines.

## Materials and methods

**Figure 1:**
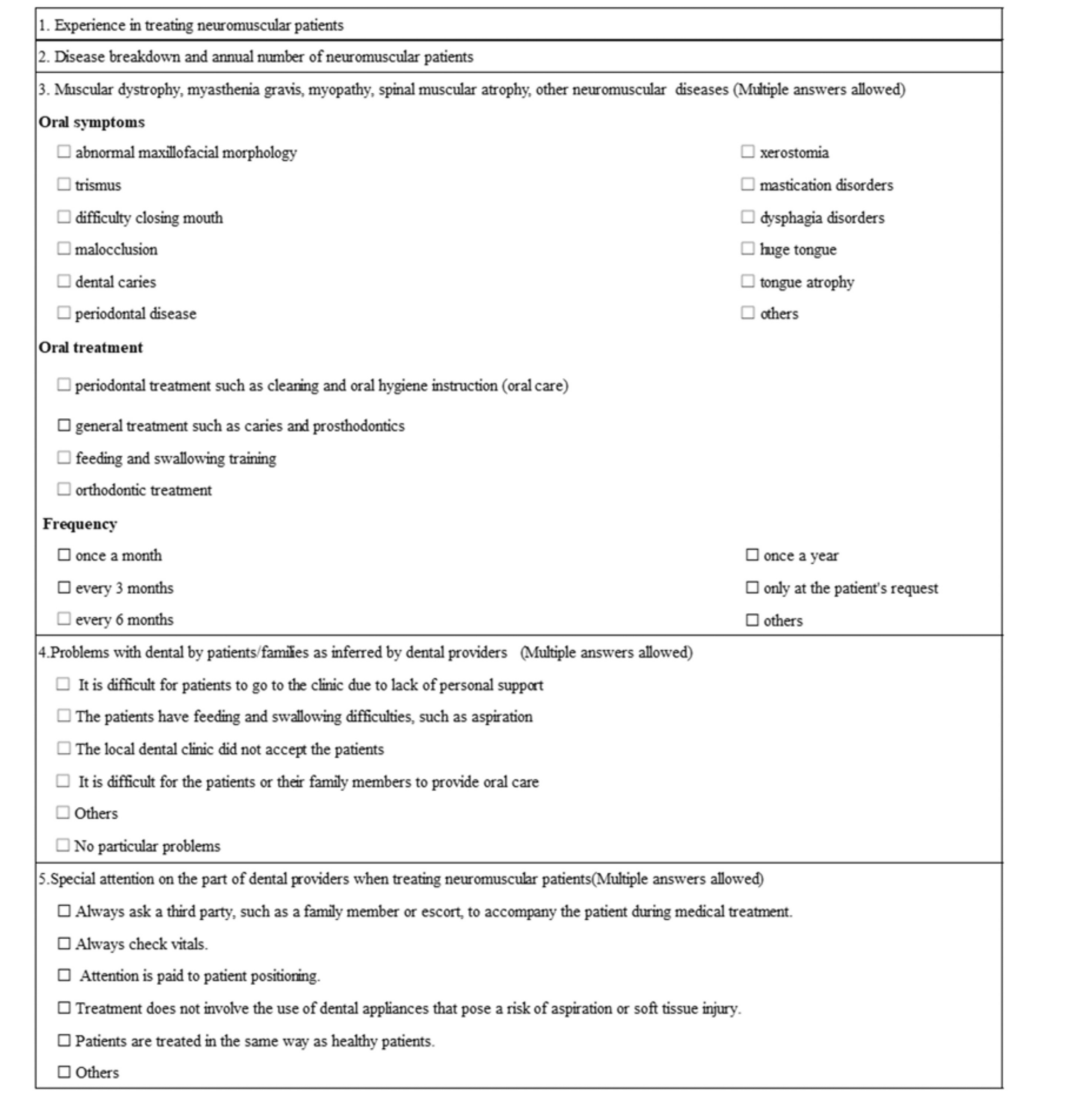
Surveyed items in the questionnaire

Between October 25 and December 31, 2021, we mailed the research purpose and questionnaire documents to dental hospitals, dental departments from university hospitals, and oral health centers and private practices that were registered as members of the Japanese Society of Disability Dentistry as of October 2021. The response method was by fax or via website, and a response was considered to provide the required informed consent. The items examined were: 1) whether or not the respondent had experience in treating patients with neuromuscular diseases; 2) the characterization of specific neurological or neuromuscular diseases, and the number of patients with each disorder treated per year; 3) the oral symptoms, treatment details, and frequency of hospital visits of patients with neuromuscular diseases (muscular dystrophy, myopathy, spinal muscular atrophy, myasthenia gravis, and other neurological or muscular disorders); 4) problems encountered by patients and their families while receiving dental treatment; and 5) points that were accorded particular attention by dental healthcare professionals when treating patients with neurological or muscular disorders (Figure 1). Questionnaires were sent to 658 facilities.

This study was conducted with the approval of the Ethics Committee for Medical Research of Juntendo University Hospital (approval number M21-024), and the answers to the questionnaires were anonymized for the analysis.

## Results

Response rate and experience of treating patients with neuromuscular diseases

Questionnaires were received from 215 facilities (96 by fax, 119 via the website). The response rate was 32.7%. Of the 215 facilities, 160 (74.4%) had experience treating patients with neuromuscular diseases.

Characterization of specific neuromuscular diseases in treated patients and the annual number of patients treated

**Figure 2:**
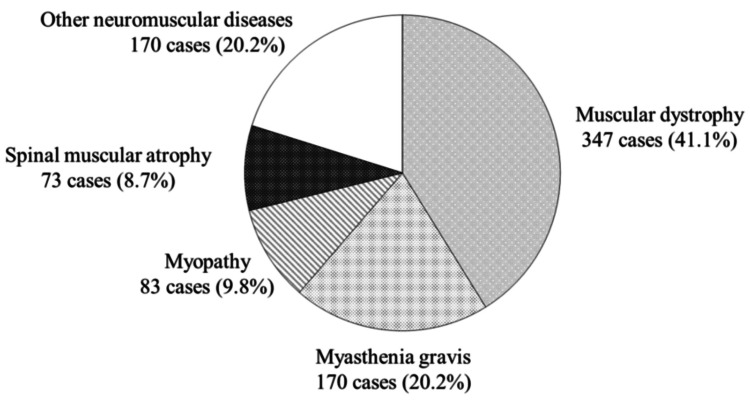
Disease breakdown and annual number of patients with neuromuscular intractable disease

The total number of patients covered by this survey was 843. There were 347 patients with muscular dystrophy (41.1%), 170 with myasthenia gravis (20.2%), 83 with myopathy (9.8%), 73 with spinal muscular atrophy (8.7%), and 170 with other neuromuscular intractable diseases (20.2%) (Figure 2). The category “Other diseases” encompassed 20 different diseases, including amyotrophic lateral sclerosis (ALS), basal ganglia degeneration, spinocerebellar degeneration, myelomeningocele, multiple sclerosis, and Parkinson's disease.

Oral symptoms, treatment content, and frequency of visits

Patients With Muscular Dystrophy

The most common oral symptom was periodontal disease (16.9%), followed by dental caries (12.7%), malocclusion (12.3%), dysphagia disorder (10.5%), and mastication disorder (10.3%). Oral hygiene, including removal of dental calculus and plaque, accounted for approximately half of the treatments (49.8%), followed by general treatment such as dental caries treatment. The frequency of visits varied depending on the patient's condition and oral symptoms, but the most common was once every three months (37.7%), followed by once a month and by visits at the request of the patient. There were also visits in collaboration with other departments during hospitalization, and visits at the request of the family doctor.

Myasthenia Gravis Patients

The most common oral symptom was periodontal disease (22.2%), followed by dysphagia and dental caries, which had the same approximate frequency (16%). Oral hygiene accounted for about half (49.1%) of the treatments. The most common frequency of visits was once every three months (40.4%), followed by those requested by the patient (32.1%). In some cases, intensive dental treatment was carried out during a hospital stay.

Myopathy Patients

The most common oral symptoms were periodontal disease (19.9%), dysphagia disorder (13.0%), and mastication disorder (12.3%). Oral hygiene accounted for over half of the treatments (51.8%), followed by general treatment (30.1%) and swallowing training (13.3%). The most common frequency of visits was every three months (39.6%), followed by visits requested by the patient (22.9%) and monthly visits (20.8%). As with patients with muscular dystrophy, there were also cases of treatment during hospitalization or at the request of a neurologist.

Patients With Spinal Muscular Atrophy

The most common oral symptom was periodontal disease (20.6%), followed by mastication disorder (13.1%) and dysphagia disorder (12.6%). Oral hygiene accounted for more than half of the treatments (54.1%), followed by dental caries treatment (31.6%) and swallowing training (10.2%). The most common frequency of hospital visits was once a month or as requested by the patient (29.0% in each case), followed by once every three months (25.8%). As with patients with muscular dystrophy, there were also cases of only hospital visits at the time of admission and hospital visits at the request of the neurologist.

Patients With Other Neuromuscular Diseases

Periodontal disease was the most common oral symptom (18.9%), followed by dysphagia disorder, dental caries, and mastication disorder (approximately 13% in all cases). In terms of treatment, oral cleaning accounted for approximately half of them (48.1%), and when combined with general treatment and swallowing training, this accounted for 98.5%. The most common frequency of visits was once every three months, followed by once a month and then at the patient's request (Table 1).

**Table 1:** Oral symptoms, treatment, and frequency of hospital visits in patients with neuromuscular intractable disease Oral symptoms in patients with neuromuscular diseases: “Others” included speech disorders, dysarthria, flow court, hypersensitivity, temporomandibular joint dislocation, bite wear, poor oral hygiene, aspiration, involuntary movements, nasopharyngeal atresia, and dystonia Oral treatment of patients with neuromuscular disease: “Others” included tooth extraction, night guard preparation, prosthetic treatment of defects, tumor resection, temporomandibular joint treatment, intraoral examination before bone resorption inhibitor treatment, follow-up after oral cancer treatment, and preoperative oral management Frequency of visits to the hospital by patients with neuromuscular disease: “Others” included that only during hospitalization, when requested by a neurologist, two to three times a week, once a week, once every one to two weeks, and once every four months

Category	Answers	Muscular dystrophy	Myasthenia gravis	Myopathy	Spinal muscular atrophy	Others
Oral symptoms	Periodontal disease	85 (16.9%)	35 (22.2%)	29 (19.9%)	36 (20.6%)	49 (18.9%)
	Dental caries	64 (12.7%)	25 (15.8%)	13 (8.9%)	20 (11.4%)	34 (13.1%)
	Malocclusion	62 (12.3%)	9 (5.7%)	15 (10.3%)	13 (7.4%)	22 (8.5%)
	Dysphagia disorder	53 (10.5%)	26 (16.5%)	19 (13%)	22 (12.6%)	35 (13.5%)
	Mastication disorder	52 (10.3%)	20 (12.7%)	18 (12.3%)	23 (13.1%)	31 (12.1%)
	Trismus	49 (9.7%)	11 (7%)	13 (8.9%)	19 (10.9%)	26 (10%)
	Huge tongue	35 (7%)	3 (1.9%)	6 (4.1%)	6 (3.4%)	8 (3.1%)
	Abnormal maxillofacial morphology	32 (6.3%)	2 (1.2%)	7 (4.8%)	8 (4.6%)	12 (4.6%)
	Occlusive mouth disorder	30 (6%)	11 (6.9%)	9 (6.2%)	10 (5.7%)	13 (5%)
	Xerostomia	26 (5.1%)	14 (8.9%)	11 (7.5%)	16 (9.1%)	20 (7.7%)
	Tongue atrophy	4 (1%)	1 (0.6%)	2 (1.4%)	1 (0.6%)	7 (2.7%)
	Others	11 (2.2%)	1 (0.6%)	4（2.7％）	1 (0.6%)	2 (0.8%)
	Total	503 (100%)	158 (100%)	146 (100%)	175 (100%)	259 (100%)
Treatment details	Oral cleaning	120 (49.8%)	53 (49.1%)	43 (51.8%)	53 (54.1%)	64 (48.1%)
	Dental caries treatment	82 (34%)	36 (33.3%)	25 (30.1%)	31 (31.6%)	46 (34.6%)
	Feeding and swallowing training	33 (13.7%)	16 (14.8%)	11 (13.3%)	10 (10.2%)	21 (15.8%)
	Orthodontic treatment	2 (0.8%)	1 (0.9%)	1 (1.2%)	1 (1%)	0 (0%)
	Others	4 (1.7%)	2 (1.9%)	3 (3.6%)	3 (3.1%)	2 (1.5%)
	Total	241 (100%)	108 (100%)	83 (100%)	98 (100%)	133 (100%)
Frequency of visits	Once a month	29 (17.9%)	9 (16.1%)	10 (20.8%)	18 (29%)	21 (30.4%)
	Every three months	61 (37.7%)	19 (40.4%)	19 (39.6%)	16 (25.8%)	29 (42%)
	Every six months	23 (14.2%)	5 (8.9%)	2 (4.2%)	2 (3.2%)	2 (2.9%)
	Once a year	6 (3.7%)	2 (3.6%)	0 (0%)	2 (3.2%)	0 (0%)
	Only at the patient's request	25 (15.4%)	18 (32.1%)	11 (22.9%)	18 (29%)	14 (20.3%)
	Others	18 (11.1%)	3 (5.4%)	6 (12.5%)	6 (9.7%)	3 (4.4%)
	Total	162 (100%)	56 (100%)	48 (100%)	62 (100%)	69 (100%)

Problems faced by patients and their families related to dental care, according to dental care workers

The most common response was that oral care was difficult for the patients and families (116 cases, 27.6%), followed by “the local dental clinic does not accept them” (24.2%), “it is difficult to go to the clinic because there is no caregiver” (23.5%), and “difficulty with eating and swallowing, such as aspiration” (21.4%) (Table 2). There were also nine responses (2.1%) indicating the absence of particular problems. Other responses included the following: difficulty in treating dental alignment and occlusion, difficulty in accessing general dentistry because the facility was not barrier-free, and not knowing where to get treatment due to a shortage of specialist dental care institutions (Table 2).

**Table 2:** Problems with dental by patients/families as inferred by dental providers

Answers	Cases
It was difficult for the patients or their family members to provide oral care	116 (27.6%)
The local dental clinic did not accept the patients	102 (24.2%)
It was difficult to go to the clinic due to the lack of personal support	99 (23.5%)
The patients had feeding and swallowing difficulties, such as aspiration	90 (21.4%)
No particular problems	9 (2.1%)
Others	5 (1.2%)

Points that dental care workers pay particular attention to when providing dental treatment for patients with neuromuscular diseases

The most common points of special care by dental healthcare professionals during treatment were as follows: positioning to prevent aspiration and ensure appropriate suction (35.2%), asking a third party to accompany the patient to the hospital (22.1%), checking the vital signs of the patient during treatment (21.9%), and avoiding the use of sharp instruments that could be dangerous to the patient (10.3%) (Table 3). In addition, 26 respondents (6.7%) reported that they treated the patients the same way as they would any other patient. Other responses included having someone else present to notice any changes when performing the treatment, always using a rubber dam and two vacuums, and being careful when talking to the patient during treatment because a mental handicap was not necessarily present (Table 3).

**Table 3:** Special attention on the part of dental providers when treating neuromuscular patients

Answers	Cases
Attention is paid to patient positioning and proper suctioning to prevent aspiration	137 (35.2%)
Always ask a third party, such as a family member or escort, to accompany the patient during medical treatment	86 (22.1%)
Always check vitals	85 (21.9%)
Treatment does not involve the use of dental appliances that pose a risk of aspiration or soft tissue injury	40 (10.3%)
Others	15 (3.8%)
Patients were treated in the same way as normal patients	26 (6.7%)

## Discussion

We conducted a survey of the current situation and problems faced by patients with neuromuscular diseases when receiving dental care at medical institutions across Japan. We examined the potential for solutions in oral function management. In May 2014, the Act on Medical Care for Patients with Intractable Diseases was promulgated in Japan, and a medical fee subsidy system for intractable diseases was launched. As of October 2023, there were 338 designated intractable diseases eligible for medical fee subsidies. Of these, 83 were neurological and muscular diseases, accounting for approximately a quarter of the total number of eligible intractable diseases [7]. Neurological and muscular diseases are those in which motor functions are impaired due to disorders of the brain, spinal cord, peripheral nerves, or muscles, and include muscular dystrophy, myopathy, Parkinson's disease, ALS, spinocerebellar degeneration, and myasthenia gravis. It is estimated that there are 180,000 patients with Parkinson's disease, 23,000 patients with spinocerebellar degeneration, 23,000 patients with muscular dystrophy, and 11,000 patients with ALS in Japan [8]. The number of patients with neuromuscular diseases is large, and it is expected that they will require dental treatment. In addition, neuromuscular diseases can cause a variety of changes over time after onset, such as a decrease in vital capacity due to progressive muscle atrophy, impaired lung growth due to spinal and thoracic deformities, and airway narrowing due to the backward movement of the lower jaw caused by skeletal muscle tension. However, there have been no cross-sectional or longitudinal studies on oral symptoms related to these diseases, and the actual situation has not yet been fully elucidated. Therefore, it is necessary to respond to oral dysfunction according to the condition of each individual.

Response rate and experience of treating patients with neuromuscular disease

We found that a large number of facilities (approximately three-quarters of those who responded to the questionnaire) had experience treating patients with neuromuscular diseases. Considering that the prevalence of muscular dystrophy is 17-20 per 100,000 individuals (0.02%) and that of myasthenia gravis is 18.6 per 100,000 individuals (0.019%) [8], it is reasonable to assume that these patients are likely to attend general dental clinics in their neighborhood, rather than dental hospitals or dental and oral surgery departments in university hospitals, where there are advocates for disability dentistry. Cases that prove difficult to treat at general dental clinics due to the progression of the disease are expected to be referred to specialist facilities.

There are many types of neuromuscular diseases, both congenital and acquired, and in this study, the questionnaire was divided into four categories for congenital neuromuscular diseases (including muscular dystrophy), moreover "others" as fifth category. Parkinson's disease is more common in adults, but in this study, the responses were obtained with these four categories, which mainly target congenital neuromuscular diseases. Therefore, it is thought that the number of patients with Parkinson's disease may have been lower than the actual number because it was entered as an item in the “others” category. In addition, Parkinson's disease is treatable, and patients can lead normal lives for approximately 10 years after the onset of the disease, making their visit to general dental clinics likely. A survey focusing on specific diseases is warranted in the future.

Oral symptoms, treatment content, and frequency of visits

Patients with neuromuscular diseases show facial muscle weakness and muscle atrophy. Muscle weakness and increased dependence on nursing care make oral care more difficult [6,8].

Muscular dystrophy is characterized by muscle damage and atrophy of the masticatory muscles, and is accompanied by severe fat tissue infiltration. It has been reported that 69.4% of patients with Duchenne muscular dystrophy have open bites and 59.7% have reversed occlusion [9,10]. In addition, abnormalities in the preparatory and oral phases, such as abnormal occlusion and macroglossia, usually appear during adolescence, causing feeding and swallowing disorders [11]. In our study, dental malocclusion, dysphagia disorder, and mastication disorder accounted for approximately one-third of the muscular dystrophy cases. Dental malocclusion makes it difficult to perform oral self-care, and dental intervention is essential. Therefore, oral cleaning was the most common treatment, accounting for 49.8% of the total number of treatments. The most common frequency of visits for cleaning was once every three months or once a month, suggesting that regular oral care is being performed to prevent oral diseases and perform eating and swallowing exercises.

Myasthenia gravis is an organ-specific autoimmune disease caused by autoantibodies against acetylcholine receptors at the neuromuscular junction. Symptoms often appear in the eyes, and dysphagia disorder, mastication disorder, and facial muscle weakness may be observed [3,12]. In our results, periodontal disease was the most common oral symptom, but dysphagia was observed in 16% of cases. Removal of dental calculus was the most common treatment. The most common frequency of hospital visits was every three months, followed by only at the patient’s request. The symptoms of this disease, which is characterized by worsening with exercise and improvement following rest, change from day to day and even within a single day. For this reason, it is highly likely that patients choose to visit the hospital during the periods of relative improvement in their symptoms, rather than following a regular schedule.

Myopathy is a general term for muscle diseases that are characterized by muscle weakness and hypotonia, which manifest from early infancy and persist thereafter [13]. Facial muscle weakness causes patients affected by this disease to exhibit a long, thin face with a limited range of facial expressions. Weakness of the orbicularis oris muscle can make it difficult to open the mouth or drink through a straw. In our study, dysphagia disorder, mastication disorder, and trismus accounted for approximately one-third of the myopathy cases. Periodontal disease was the most common oral symptom. In addition to dysphagia, mastication disorder, and trismus, malocclusion and xerostomia were also observed, which were attributed to periodontal disease. As with muscular dystrophy, it was inferred that dentists regularly carried out dental intervention and oral management from infancy, while feeding and swallowing training was also performed.

Spinal muscular atrophy is an autosomal recessive genetic disease classified according to onset time [14]. Progressive muscle weakness and muscle atrophy of the limbs, pharynx, and trunk are common to all types of the disease. In this study, periodontal disease was the most common problem in these patients. This is likely caused by the inability of patients to brush their teeth properly every day due to muscle weakness in their limbs, relying instead on hospital visits once a month for oral care. The number of visits at the request of the patient was almost the same as the number of scheduled monthly visits. Therefore, it is possible that they only visited the dentist when they experienced oral symptoms.

Patients with neuromuscular diseases are most likely to suffer from periodontal disease. In neuromuscular diseases, abnormalities of the jaw and face can occur due to muscle atrophy or muscle disease. In addition, accumulation of food residues in the mouth due to dysphagia disorder or mastication disorder, macroglossia, reduced self-cleansing due to the atrophy of the tongue, reduced oral function, and xerostomia. These increase the difficulty in self-care, which induces poor oral hygiene. As a result, patients may develop dental diseases. However, dental treatment may improve their quality of life. Furthermore, it is expected that patients who have received dental treatment will visit the dental clinic once every three months or once a month to continue oral management on a regular basis.

Problems for patients and families receiving dental treatment

Even if patients with oral conditions that require dental intervention wish to visit the dentist, there are cases where they cannot be treated at a nearby general practice or transferred to a dental chair. Patients with neuromuscular diseases present with symptoms specific to their condition, and the degree of disability varies depending on the stage and condition of the disease, making treatment even more complex. Dentists who treat many patients with neuromuscular dystrophy hope local dentists will accept these patients. However, local dentists may have little opportunity to treat patients with neuromuscular diseases and may not be very interested in doing so [15]. It is hoped that Japanese dental hospitals that advocate dental care for people with disabilities, as well as oral surgery departments at university hospitals, will take the lead in educating local dental care professionals about the importance of dental intervention for patients with these diseases, and promote regional cooperation between dentists and dental and oral surgery departments. In addition, some respondents reported that there were no particular problems associated with treating patients with these diseases, suggesting that general dentists could treat patients with mild symptoms in an outpatient setting.

Makizodila et al. [6] reported that 71.9% of 259 respondents to an online survey of patients with neuromuscular disorders mentioned that they had not been informed of the importance of maintaining oral health, 40.4% that they would have liked to receive help from oral healthcare specialists regarding oral hygiene, and 19.8% that they were not satisfied with the oral care they received either for themselves or from their caregivers. We concluded that the oral hygiene of patients with neuromuscular diseases requires attention from both multidisciplinary treatment teams and oral care specialists, and that this is a problem that is not necessarily restricted to Japan.

Special considerations for dental professionals in the treatment of patients with neuromuscular diseases

Patients with neuromuscular disorders have difficulty maintaining a sitting position due to involuntary movements and muscle weakness, and therefore, special consideration is required when positioning them for dental treatment. In addition, because swallowing and breathing are restricted during dental treatment, appropriate suctioning is necessary, especially for patients with dysphagia or respiratory dysfunction. Moreover, the measurement of vital signs is important in all invasive dental treatments. This is especially crucial during procedures that involve a risk of bleeding, such as extractions or minor oral surgery. It is necessary to work closely with the professionals who are in charge of patient management to obtain sufficient functional evaluation and information before starting dental treatment, and to understand the patient's condition and the progression of the disease in order to provide safe dental treatment. In addition, even for patients with neuromuscular disorders who are able to receive standard treatment, it is necessary to carry out oral management that considers the probable progression of their condition in the future.

In this study, the age of the patients was not recorded, and the degree of progression of their condition at the time of their visit to the hospital is unknown. However, by providing dental intervention from childhood, the dentist can monitor the patient's condition over time and build a relationship of trust. We believe that it is necessary to educate patients about the importance of oral management from childhood. Treating these patients requires special care and appropriate medical staff, equipment, and knowledge about the specific disease affecting them. In order to address these issues, it is urgently necessary to collaborate with medical and specialized dental facilities and to receive support from the government.

Limitations of the study

One limitation of this study was the low response rate. This low response rate may be due to the survey being conducted during the coronavirus pandemic. The questionnaire was sent by standard mail, but insufficient reminders may have contributed to the low response rate. This issue should be considered in future studies.

## Conclusions

Many patients with neuromuscular diseases have several dental problems, such as reduced mastication and dysphagia disorder and difficulties with self-care, and require the intervention of a dentist. Patients and their dentists hope to be accepted by local dentists, but it has been found that many dental clinics do not have the facilities or staff to accept these patients.

In addition to deepening their knowledge of these conditions, dentists need to collaborate with medical institutions and specialist dental facilities, and it is considered that urgent action is needed to provide administrative support and create guidelines.
